# Development of NAFLD‐Specific Human Liver Organoid Models on a Microengineered Array Chip for Semaglutide Efficacy Evaluation

**DOI:** 10.1111/cpr.70118

**Published:** 2025-08-27

**Authors:** Xiao‐yan You, Xiang‐yang Li, Hui Wang, Guo‐ping Zhao

**Affiliations:** ^1^ Henan Engineering Research Center of Food Microbiology, College of Food and Bioengineering Henan University of Science and Technology Luoyang China; ^2^ Master Lab for Innovative Application of Nature Products, National Center of Technology Innovation for Synthetic Biology Tianjin Institute of Industrial Biotechnology, Chinese Academy of Sciences (CAS) Tianjin China; ^3^ Haihe Laboratory of Synthetic Biology Tianjin China; ^4^ School of Life Sciences, Faculty of Medicine, Tianjin Key Laboratory of Function and Application of Biological Macromolecular Structures Tianjin University Tianjin China; ^5^ CAS‐Key Laboratory of Synthetic Biology, CAS Center for Excellence in Molecular Plant Sciences Institute of Plant Physiology and Ecology, Chinese Academy of Sciences Shanghai China; ^6^ CAS Key Laboratory of Quantitative Engineering Biology Shenzhen Institute of Synthetic Biology, Shenzhen Institute of Advanced Technology, Chinese Academy of Sciences Shenzhen China; ^7^ Engineering Laboratory for Nutrition Shanghai Institute of Nutrition and Health, Chinese Academy of Sciences Shanghai P. R. China

**Keywords:** array chip, drug assessment, non‐alcoholic fatty liver, organoid, semaglutide

## Abstract

Progressive non‐alcoholic fatty liver disease (NAFLD) may culminate in severe complications, including fibrosis, cirrhosis and hepatocellular carcinoma, yet therapeutic breakthroughs remain elusive, necessitating novel pharmacological strategies. Semaglutide, a glucagon‐like peptide‐1 (GLP‐1) receptor agonist clinically approved for type 2 diabetes and obesity management, has demonstrated pleiotropic effects in preclinical NAFLD models. In this study, we investigated semaglutide's therapeutic efficacy and mechanisms in a human liver organoids (hLOs) model of NAFLD. Utilising microengineered array chips, human induced pluripotent stem cells (hiPSCs) were differentiated into hLOs with functional hepatic properties. NAFLD pathology was induced via free fatty acid (FFA) exposure, recapitulating disease hallmarks such as steatosis, inflammatory cytokine elevation and fibrogenic activation. Semaglutide treatment at 50 nM significantly attenuated lipid deposition caused by FFAs and reduced triglyceride levels by 8‐fold and cholesterol levels by 1.8‐fold. It also inhibited the expression of pro‐inflammatory markers (IL‐6, IL‐8, TNF‐α) by about 1.5–2 fold and increased the level of lipolytic genes by about 45%. These findings elucidate the therapeutic potential of semaglutide in attenuating key NAFLD‐associated pathologies and establish a robust in vitro platform for preclinical drug evaluation. The study provides critical insights into targeted NAFLD interventions and supports the translation of GLP‐1‐based therapies into clinical practice, addressing an unmet need in hepatology.

## Graphical abstract

Microporous array organ chips were integrated with commercially available well plates to develop organoid chip platforms, which enable modelling of hepatic physiology and non‐alcoholic fatty liver disease (NAFLD) pathogenesis, as well as evaluation of semaglutide therapeutics.

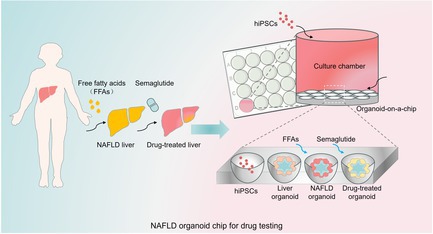

## 1 Introduction

Non‐alcoholic fatty liver disease (NAFLD) has emerged as a critical global health challenge, affecting one in four individuals worldwide—a concerning statistic driven by rising obesity rates and metabolic disorders [1, 2]. Characterised by excessive fat deposition in the liver, NAFLD is not merely a benign condition but a stealthy precursor to life‐threatening complications, including fibrosis, cirrhosis and hepatocellular carcinoma [3, 4, 5]. Despite its alarming prevalence, therapeutic breakthroughs remain elusive, leaving millions without effective treatment. This unmet clinical urgency underscores the imperative to redefine how we study and combat NAFLD—a challenge demanding innovation at the intersection of pharmacology and biotechnology.

Semaglutide, a glucagon‐like peptide‐1 (GLP‐1) receptor agonist with dual glycaemic control and weight‐loss efficacy shown tantalising promise in alleviating NAFLD symptoms since its 2021 FDA approval for weight management, with clinical trials reporting reduced liver fat, improved metabolic markers and attenuated inflammation [6, 7, 8]. Yet the path from lab to clinic is fraught with hurdles: traditional animal models, while invaluable, often fail to predict human responses due to inherent biological differences [9, 10, 11]. For instance, studies in NAFLD mice reveal semaglutide's ability to curb weight gain and oxidative stress [11, 12], but translating these findings to humans remains a gamble. This translational gap highlights the need for models replicating human NAFLD pathophysiology.

Human induced pluripotent stem cell (hiPSC) derived hLOs have become a transformative tool for modelling liver physiology and disease, bridging the gap between 2D monocultures and animal models. In recent years, advances in directed differentiation technology have enabled the gradual induction of differentiation of hiPSCs into hLOs with higher cellular heterogeneity and functional maturity by manipulating the concentration of specific factors [13]. Currently, there are two main ways of hiPSCs‐derived hLOs. The first one is to induce hiPSCs to differentiate into mature hepatocytes under 2D culture and subsequently generate hLOs by 3D culture. The second one is to further differentiate hiPSCs into mature hLOs directly under 3D culture conditions, after hiPSCs become spheroids with the help of self‐assembly ability [14, 15]. Both ways can successfully generate hLOs and maintain the characteristics of the liver in long‐term culture.

Suspension culture is the most commonly used traditional method for organoid culture, whereby spheres are formed by means of ultra‐low adsorption microtiter plates with the help of the self‐assembling ability of hiPSCs [15]. However, it has to be said that current organoid platforms lack spatial control over organoid formation and often uncontrolled fusion leads to central necrosis and heterogeneity of drug response, a limitation that hampers their use in preclinical pharmacological studies. Microengineering technology enables the construction of array chips with predefined pore sizes and spacing through micron‐level precision material processing (e.g., soft lithography, etc.), which confines cells in separate pores to form homogeneous organoid spheres and addresses the limitations of traditional organoid culture [16]. Based on this, the organoid chip technology offers a transformative solution. By integrating stem cell‐derived organoids with microengineered perfusion systems, these platforms recreate miniature, functional human tissues in vitro, complete with dynamic fluid flow and 3D architecture [17, 18, 19, 20]. Pioneering work has already demonstrated their potential: liver organoids exposed to fatty acids replicate NAFLD's hallmarks—steatosis, inflammation and fibrosis [21], while lobule‐mimicking chips reveal spatial patterns of lipid accumulation and drug response [22]. Such advances hint at a future where drug candidates like semaglutide can be tested in environments that closely resemble human physiology, bypassing the limitations of animal trials. Nevertheless, despite these advancements, the therapeutic mechanisms of semaglutide in NAFLD remain unexplored. Here, we constructed microporous array chips based on soft lithography for the in situ formation of hiPSCs‐derived hLOs. Meanwhile, free fatty acid (FFA) exposure successfully induced NAFLD. Based on this platform, we evaluated the therapeutic activity of semaglutide for NAFLD.

## 2 Experiment and Methods

### 2.1 Preparation of Microporous Array Chip

The microporous array chip was fabricated from polydimethylsiloxane (PDMS) using soft lithography [23, 24, 25]. The chip architecture was engineered to incorporate a microporous array (600 μm diameter, 50 μm gap, 600 μm height), enabling the in situ, high‐throughput production of hLOs within the wells. The PDMS prepolymer and curing agent (Dow Corning Corp) were combined in a 10:1 ratio and cast into the mold. Residual gas bubbles were eliminated via degassing with a circulating water vacuum pump, followed by thermal curing in an oven (PH‐030A) at 90°C for 1 h. Then, the chips were sterilised in an autoclave (SX‐700), combined with commercial 24‐well plates (Corning, 5201) and exposed to UV light (SW‐CJ‐IFD) for 4–6 days. Prior to experimentation, the chip surface was functionalised for hydrophobicity by treatment with 0.2% (w/v) PF127.

### 2.2 hiPSCs Culture and Expansion

hiPSCs were cultured as previously described [23]. hiPSC cells (DYR0100) from the Chinese scientific cell bank. Matrigel (BD Biosciences, 356234) was reconstituted in DMEM/F12 medium (Gibco, C11330500BT) at 1:50 (v/v) and coated onto 6‐well plates for 4 h at 4°C. After equilibration in mTeSR1 medium (StemCell, 85850) at 37°C for 1 h, hiPSCs were seeded at 1–2 × 10^4^ cells/cm^2^. Cultures were maintained in mTeSR1 with daily medium changes. At 80%–90% confluence, cells were enzymatically dissociated using Accutase (Sigma‐Aldrich, A6964) and passaged at a 1:6 split ratio. Post‐dissociation, cells were incubated in mTeSR1 supplemented with 10 μM Y27632 (Selleck, S1049) for 1 h to enhance viability.

### 2.3 Liver Organoid Construction in Array Chips

The method was adopted according to the previous study [26], hLOs differentiation followed a four‐stage protocol (Figure S1A). hiPSCs were dissociated and transferred to array chips in mTeSR1 medium containing 10 μM Y27632, where embryoid bodies (EBs) formed overnight. Stage‐specific media formulations were: Days 0–5 (Definitive endoderm): RPMI 1640 supplemented with 2% KnockOut Serum Replacement (KSR), 1× B27, 1× GlutaMAX, 1% penicillin–streptomycin and 100 ng/mL Activin‐A. Days 5–10 (Hepatic progenitors): Activin‐A replaced with 20 ng/mL bFGF and 10 ng/mL HGF. Days 10–15 (Hepatocyte maturation): Hepatocyte Culture Medium (HCM) containing 20 ng/mL oncostatin M (OSM) and 1 μM dexamethasone (Dex). Days 15–20: HCM with 1 μM Dex alone. hLO maturation was monitored daily via brightfield microscopy. Reagent details are provided in Table S1.

### 2.4 Modelling NAFLD on Array Chips

On Day 18, hLOs were supplemented with a 2:1 (v/v) mixture of oleic acid (OA, 400 μM; Sigma‐Aldrich, O7501) and palmitic acid (PA, 200 μM; Sigma‐Aldrich, P9767) for 48 h to induce steatosis. Control hLOs were maintained in normal medium containing fatty acid‐free bovine serum albumin (BSA; Sigma‐Aldrich, SRE0098). During the drug validation phase, the NAFLD model was replaced with normal culture medium for 2 days after 2 days of FFA exposure.

### 2.5 Drug Interventions

FFA‐exposed hLOs were administered semaglutide (20, 50, 100 nM; MedChemExpress, HY‐114118) or obeticholic acid (5 μM; Sigma‐Aldrich, SML3096) for 48 h. Parallel control groups included untreated physiological and pathological hLOs.

### 2.6 Immunohistochemical Staining

Spheroids harvested at each differentiation stage were fixed in 4% paraformaldehyde (PFA; Solarbio, P1110) for 30 min at room temperature, rinsed three times with Dulbecco's phosphate‐buffered saline (DPBS; Solarbio, CX‐D1040), and cryoprotected overnight at 4°C in 30% sucrose (Sigma‐Aldrich, V900116). Samples were subsequently embedded in optimal cutting temperature (OCT) compound (SAKURA, 4583), flash‐frozen in liquid nitrogen, and stored at −80°C. Cryosections (10 μm thickness) were prepared using a cryostat microtome (CM1850) and air‐dried for 1 h prior to staining.

For immunofluorescence, sections were permeabilised with 0.25% Triton X‐100 (Sigma‐Aldrich, T8787) in DPBS for 5 min, followed by blocking in 10% (v/v) goat serum (Solarbio) for 1 h at room temperature. Primary antibodies (dilutions specified in Table S2) were applied and incubated overnight at 4°C. After three DPBS washes, sections were incubated with species‐matched Alexa Fluor‐conjugated secondary antibodies (CST, 4412s/8889s/4408s; Beyotime, A0502) for 1 h at 25°C. Nuclei were counterstained with 1 μg/mL DAPI (Cell Signalling, 4083) for 10 min. Slides were mounted in 50% glycerol/DPBS (v/v) and imaged using a DM5000B fluorescence microscope.

For lipid droplet visualisation, sections were blocked with 5% bovine serum albumin (BSA; Solarbio, CX‐A8010) and 0.25% Triton X‐100 in DPBS, then stained with 10 μM BODIPY 493/503 (MedChemExpress, 121207‐31‐6) for 1 h (protected from light). Oil Red O staining (Sigma‐Aldrich, O0625) was performed by incubating sections in filtered Oil Red O solution for 30 min, followed by three 5‐min washes in 60% isopropanol. Glycogen content was assessed via periodic acid–Schiff (PAS) staining (Solarbio, G1281), while haematoxylin and eosin (H&E; Solarbio, G1121) staining was employed for histological evaluation. All brightfield and fluorescence images were acquired using a DM5000B microscope under standardised illumination conditions.

### 2.7 Real‐Time Quantitative PCR Analysis and Transcriptomics Analysis

Total RNA was isolated from hiPSCs and hLOs using RNAiso PLUS (TaKaRa) and reverse‐transcribed into cDNA with PrimeScript RT Premix (TaKaRa) under the following conditions: 37°C for 15 min, 85°C for 5 s and 4°C hold. qRT‐PCR was performed on a LightCycler 96 System (Roche) using 40 cycles of 95°C for 30 s (denaturation), 58°C for 45 s (annealing) and 72°C for 30 s (extension). GAPDH served as the endogenous control; primer sequences are listed in Table S3.

For transcriptomic analysis, RNA from FFA‐treated hLOs (FFA‐hLOs) and semaglutide‐treated hLOs (M‐Sem‐hLOs) was extracted as above and subjected to paired‐end 150 bp sequencing on a BGISEQ‐500 platform (BGI, Shenzhen, China). *N* = 3 replicates.

### 2.8 Cell Live‐Dead Analysis

hLOs viability was assessed on Day 20 using a Live/Dead Cell Staining Kit (APExBIO, K2081). Organoids were washed with DPBS, incubated with calcein‐AM (live) and propidium iodide (dead) dyes for 30 min at 37°C, and imaged in situ using a fluorescence microscope (Leica DM5000B).

### 2.9 Biochemical Level Analysis

Media collected on Days 10 and 20 were analysed for albumin (USCN, CEB028H) and urea (BioAssay Systems, DIUR‐100) secretion. For lipid quantification, hLOs were lysed via ultrasonication (Scientz JY92‐11DN), and total cholesterol (TC; Nanjing Jiancheng, A110‐11) and triglycerides (TG; Nanjing Jiancheng, A111‐1‐1) were measured using enzymatic assays. Protein normalisation was performed via BCA assay (Lablead, B5001).

### 2.10 Statistical Analysis

Data are expressed as mean ± standard deviation (SD). Differences between two groups were evaluated using unpaired Student's *t*‐test. Significance thresholds were set at *p < 0.05, **p < 0.01 and ***p < 0.001. The data were processed with software Excel and GraphPad Prism 9.

## 3 Results and Discussion

### 3.1 Construction of an In Vitro Liver Organoid Culture Platform

**FIGURE 1:**
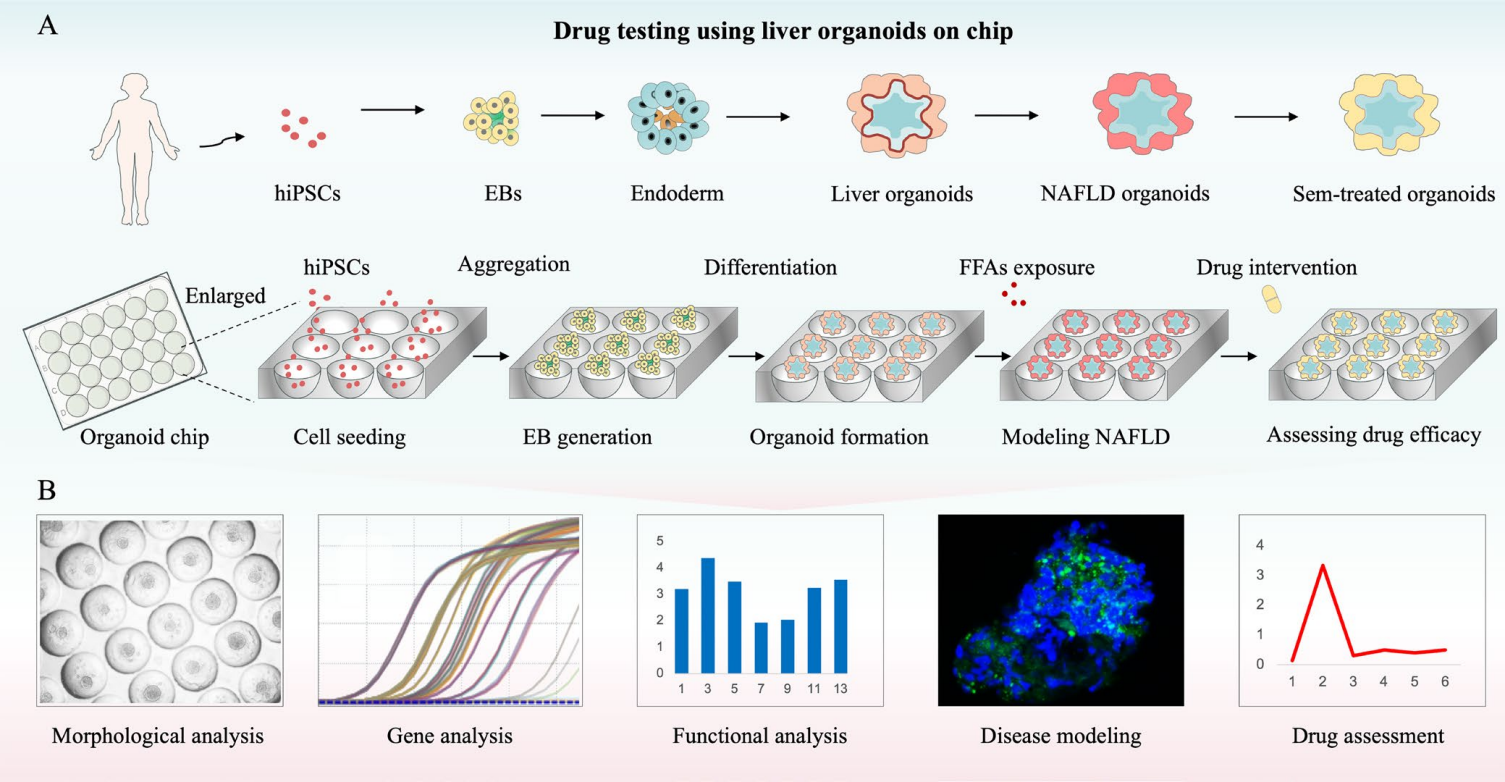
Liver organoid chip platform for semaglutide efficacy assessment in NAFLD. (A) Differentiation of hiPSCs into liver organoids within microwell arrays, enabling NAFLD modelling and semaglutide evaluation. (B) Analytical methods for phenotypic and functional assessment of organoids.

Stem cells leverage their multipotent differentiation and self‐assembly capabilities to generate diverse germ layer lineages under specific induction conditions. These lineages subsequently develop into tissue‐specific progenitor cells, ultimately forming organoids [24]. This remarkable differentiation potential enables the simulation of complex human tissue microenvironments and functional properties. As shown in Figure 1A, we induced the generation of hiPSC‐derived hLOs to model NAFLD. Although current commercially available low adsorption plates or multiwell array plates are capable of organoid construction, they limit organoid pathology studies due to problems such as the inability to control the size or high price. Herein, we designed a microtiter array chip compatible with commercial well plates, enabling the creation of a high‐throughput organoid culture system. The modular design allows customisation of microtiter parameters (number, spacing, depth, size) to accommodate varying experimental throughput or organoid size requirements. Meanwhile, the main material of the chip is selected as biocompatible PDMS, which supports long‐term cell culture as well as growth status tracking. Using this organoid‐on‐a‐chip platform, we evaluated the therapeutic efficacy of semaglutide against NAFLD. As shown in the enlarged image, the microporous array facilitates the controlled formation of 3D spheroids, supports in situ differentiation and maturation of hLOs, and enables long‐term culture (Figure 1A).

Before organoid formation, stem cells were enzymatically digested and dissociated into single‐cell suspensions, which were then seeded into well plates containing microporous array structures overnight (Figure 1A). Cultivation in microporous structures reduced the high variability in size of embryoid bodies (EBs) during growth and differentiation. Compared to conventional suspension culture, the defined shape and spacing of the microwells effectively prevented the fusion of adjacent spheroids, thereby avoiding central necrosis caused by oversized aggregates [27]. Consequently, spheroids cultured on this organoid‐on‐a‐chip platform are highly suitable for physiological and pathological modelling, enabling in situ tracking and real‐time imaging. The platform supports both on‐chip functional assays (e.g., real‐time imaging) and off‐chip analytical techniques (e.g., qPCR, ELISA, RNA sequencing) for comprehensive system evaluation (Figure 1B). In summary, the microporous array design offers a straightforward and efficient platform for controlling the differentiation, formation and functional assessment of liver organoids.

### 3.2 In Situ Formation of hiPSCs‐Derived Liver Organoid in Microwell Arrays

Generally, the differentiation of hiPSCs into liver cells involves three key stages: endoderm induction, hepatic progenitor cell differentiation and expansion, and hepatocyte maturation [27]. In this study, undifferentiated hiPSCs were directly seeded onto microwell array chips. Endoderm induction, hepatic progenitor cell expansion and liver maturation were sequentially achieved by adding stage‐specific growth factors (Figure S1A). As shown in Figures S2 and 2A, dissociated stem cells aggregated into spherical structures overnight within the microwells, gradually completing the induction of hLOs. During this process, the size of the hLOs progressively increased (Figure S1B). In addition, we investigated the formation of EBs under the same conditions using low‐adhesion 96‐well plates. Following overnight incubation, we conducted bright field observation and size analysis of the generated spheres (Figure S2B,C). Compared with the microporous array chips, the EBs generated in the low‐adhesion plates exhibited greater variability in size and demonstrated a tendency to aggregate or fuse with one another. This suggests that the microporous array chips provide a more controlled and uniform environment for EBs formation. Meanwhile, we analysed the cell viability statistics of hLOs on Day 20 using dead‐viable staining and found that hLOs had high activity, indicating a good culture status of hLOs (Figure S1C). In addition to this, in order to explore the chip biocompatibility or potential toxicity to the cells, we performed immunofluorescence staining for the apoptosis marker active protease Caspase 3 on frozen sections of hLOs on Day 20 and did not observe extensive cell death within the liver organoids. Thus, culture in this chip ensures the efficient formation of hLOs and avoids core cell necrosis (Figure S3).

**FIGURE 2:**
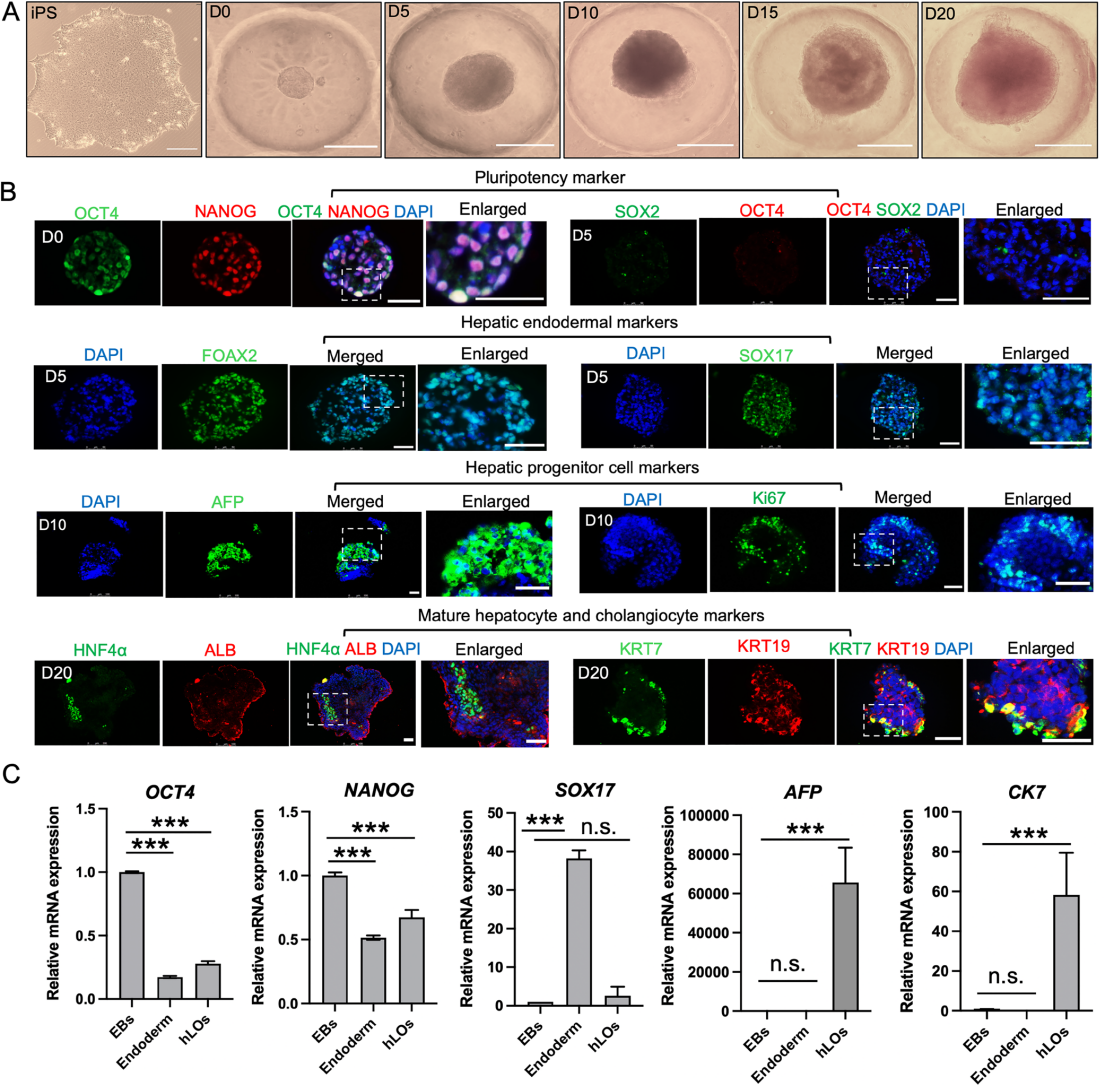
In situ formation of hLOs from hiPSCs in chip systems. (A) Brightfield micrographs of hLO differentiation stages. Scale bars: 250 μm. (B) Immunofluorescence staining of frozen sections of spheroids at different stages for characterisation of specific markers: Pluripotency (OCT4, NANOG), definitive endoderm (FOXA2, SOX17), hepatic progenitors (AFP, Ki67), mature hepatocytes (ALB, HNF4α) and cholangiocytes (KRT7, KRT19). Scale bars: 50 μm. (C) qRT‐PCR analysis of pluripotency (*OCT4*, *NANOG*), endodermal (*SOX17*), hepatic progenitor (*AFP*) and cholangiocyte (*CK7*) genes across differentiation stages (EBs, endoderm, hLOs). Data represent mean ± SD from three independent replicates (***p < 0.001 versus EBs stage).

To determine the localization and distribution of stem cell lineage markers at different stages within hLOs, immunohistochemical analyses were performed at various time points. As shown in Figure 2B, the stemness markers OCT4 and NANOG were highly expressed on Day 0, indicating that the cells remain in an undifferentiated state at the anaphase stage, with the potential for self‐renewal and differentiation into various types of cells. However, on Day 5, the stemness markers OCT4 and SOX2 were barely expressed, suggesting that the stem cells were overgrown towards the endodermal lineage. On Day 5, endodermal spheroids were identified by immunostaining for FOXA2 and SOX17, confirming the onset of endodermal differentiation. By Day 10, liver progenitor cells had differentiated, as evidenced by the expression of the AFP marker, and the cell proliferation marker Ki67 was also highly expressed. Following hepatic maturation, hLOs on Day 20 abundantly expressed mature hepatocyte‐specific markers, including ALB and HNF4α. Notably, the organoids also showed positive expression of the cholangiocyte markers KRT7 and KRT19, resembling bile duct structures in the final stage. These findings demonstrate cellular heterogeneity within the liver organoids, comprising both hepatocytes and ductal structures (Figure 2B). In addition, the expressions of ALB, HNF4a, KRT7 and KRT19 were quantitatively analysed for the percentage of ALB+, HNF4a+, KRT7+ and KRT19+ cells in liver organoids (Figure S4).

Additionally, to further characterise the differentiation and formation of hLOs, gene expression profiles representing pluripotent and hepatic lineages were analysed using real‐time PCR. Spheroids from different stages of differentiation (embryoid bodies, endoderm and liver‐like organoids) were collected, with gene expression in the embryoid body (EB) stage serving as the control (Figure 2C). The results revealed a clear trend: pluripotency markers (*OCT4* and *NANOG*) decreased as organoid differentiation progressed, while the endodermal marker *SOX17* was highly expressed. At the liver‐like organoid stage, the expression of the hepatic precursor gene AFP and the cholangiocyte marker *CK7* was significantly upregulated. These findings validate the stepwise differentiation of hiPSCs into liver organoids. In summary, the comprehensive analysis of these data demonstrates that our system effectively supports the physiological induction of liver organoids.

### 3.3 Functional Characterisation of Liver Organoids Formed on a Chip

**FIGURE 3:**
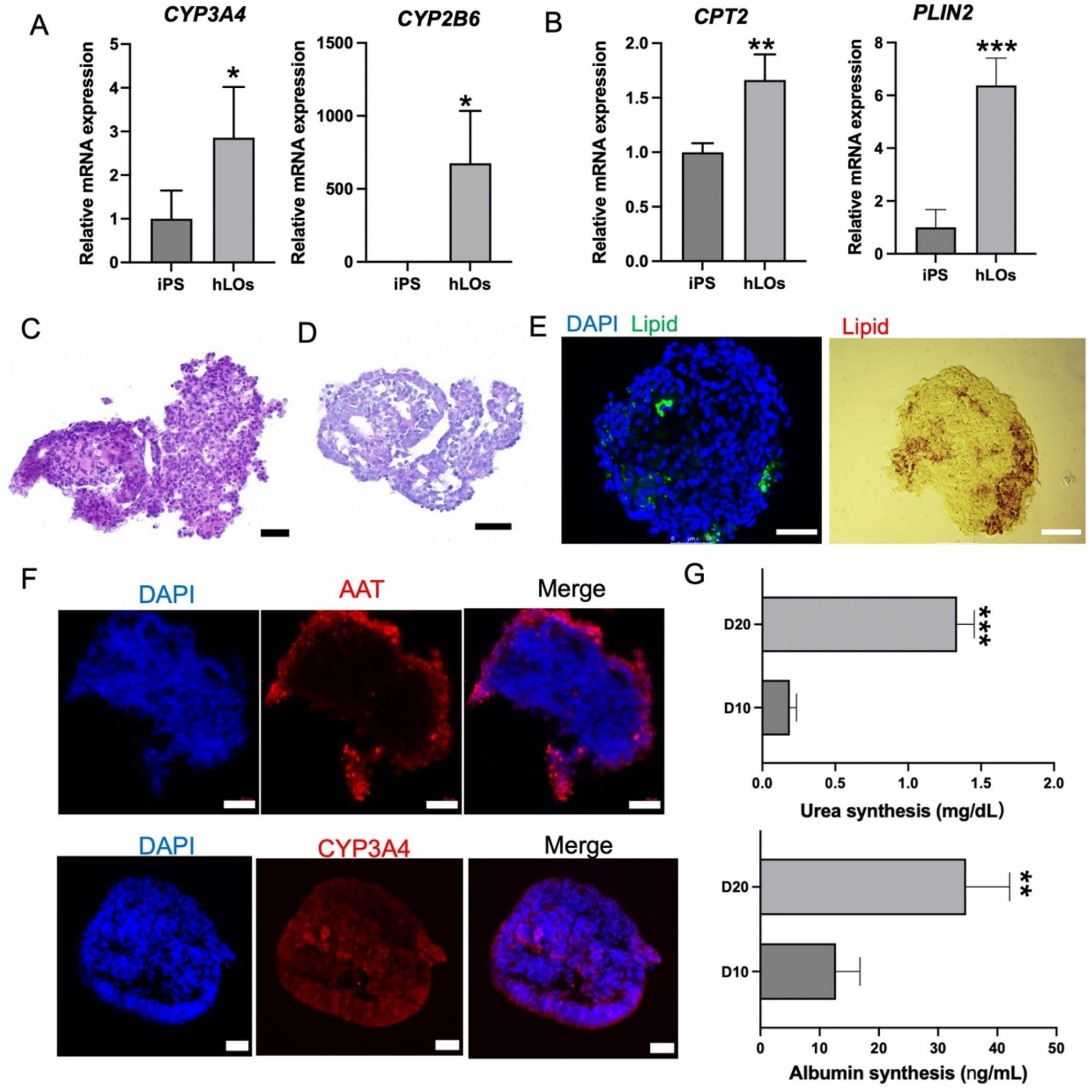
Functional Characteristics of Liver Organoids. (A) Real‐time PCR analysis of CYP450 enzyme (*CYP3A4*, *CYP2C9*) and (B) lipid metabolism gene (*CPT2*, *PLIN2*) expression in hLOs vs. hiPSCs. (C) H&E‐stained hLOs sections assessing tissue structure and cellular morphology. (D) PAS staining demonstrating glycogen storage. (E) Oil Red O and BODIPY 493/503 staining of lipid droplets. (F) Frozen section pf hLOs on Day 20 were stained for AAT and CYP3A4 to assess liver maturation and drug metabolism function. (G) Albumin secretion and urea synthesis in Day 10 versus 20 hLOs. Scale bars: 50 μm (C, D, E, F). Data represent mean ± SD from three independent replicates (*p < 0.05, **p < 0.01, ***p < 0.001).

The liver, as the largest internal organ, performs critical physiological functions including glycogen synthesis, lipid metabolism, drug detoxification (mediated by cytochrome P450 enzymes), and synthesis of serum proteins such as albumin [28]. To evaluate the functional maturation of hLOs generated on‐chip, we conducted a series of analyses. First, we examined the expression of liver‐specific enzymes and genes associated with lipid metabolism regulation using real‐time PCR. As shown in Figure 3A, the expression levels of cytochrome P450 enzymes (*CYP3A4* and *CYP2B6*) were significantly higher in the hLOs group compared to the undifferentiated hiPSCs group. Additionally, the expression of carnitine palmitoyltransferase 2 (*CPT2*) and perilipin 2 (*PLIN2*), two key markers involved in lipid metabolism, was markedly upregulated in the hLOs group relative to the hiPSCs group (Figure 3B). To assess the histological integrity and structural organisation of the hLOs, we performed haematoxylin and eosin (H&E) staining on frozen sections (Figure 3C). The results revealed well‐preserved tissue architecture and cellular morphology, indicative of mature liver‐like structures. Periodic acid‐Schiff (PAS) staining was employed to evaluate glycogen storage capacity, demonstrating the ability of hLOs to synthesise and store glycogen (Figure 3D). Furthermore, lipid accumulation within the hLOs was confirmed using Oil Red O staining and BODIPY fluorescence staining, highlighting their capacity for lipogenesis (Figure 3E). α‐1 antitrypsin (AAT) is one of the important proteins synthesised and secreted by the liver, and its expression level can reflect the maturity of liver organoids and the integrity of liver function. CYP3A4 is an important enzyme system responsible for drug metabolism in the liver. Here, we performed immunofluorescence staining on frozen sections of hLOs on Day 20 and confirmed the strong expression of ATT and CYP3A4, suggesting that hLOs have hepatocyte function and can be used for drug metabolism studies (Figure 3F). In addition to these histological and metabolic assessments, we quantified the secretion of albumin and urea as functional indicators of liver maturation. On Day 20, hLOs exhibited significantly higher levels of albumin and urea production compared to Day 10 spheroids, reflecting enhanced functional maturity (Figure 3G). Collectively, these findings demonstrate that the microwell array‐based organoid culture system effectively supports the differentiation of hLOs into multicellular structures with mature hepatic functions.

### 3.4 Construction of a Non‐Alcoholic Fatty Liver Model and Screening of the Effect Concentration of Semaglutide

**FIGURE 4:**
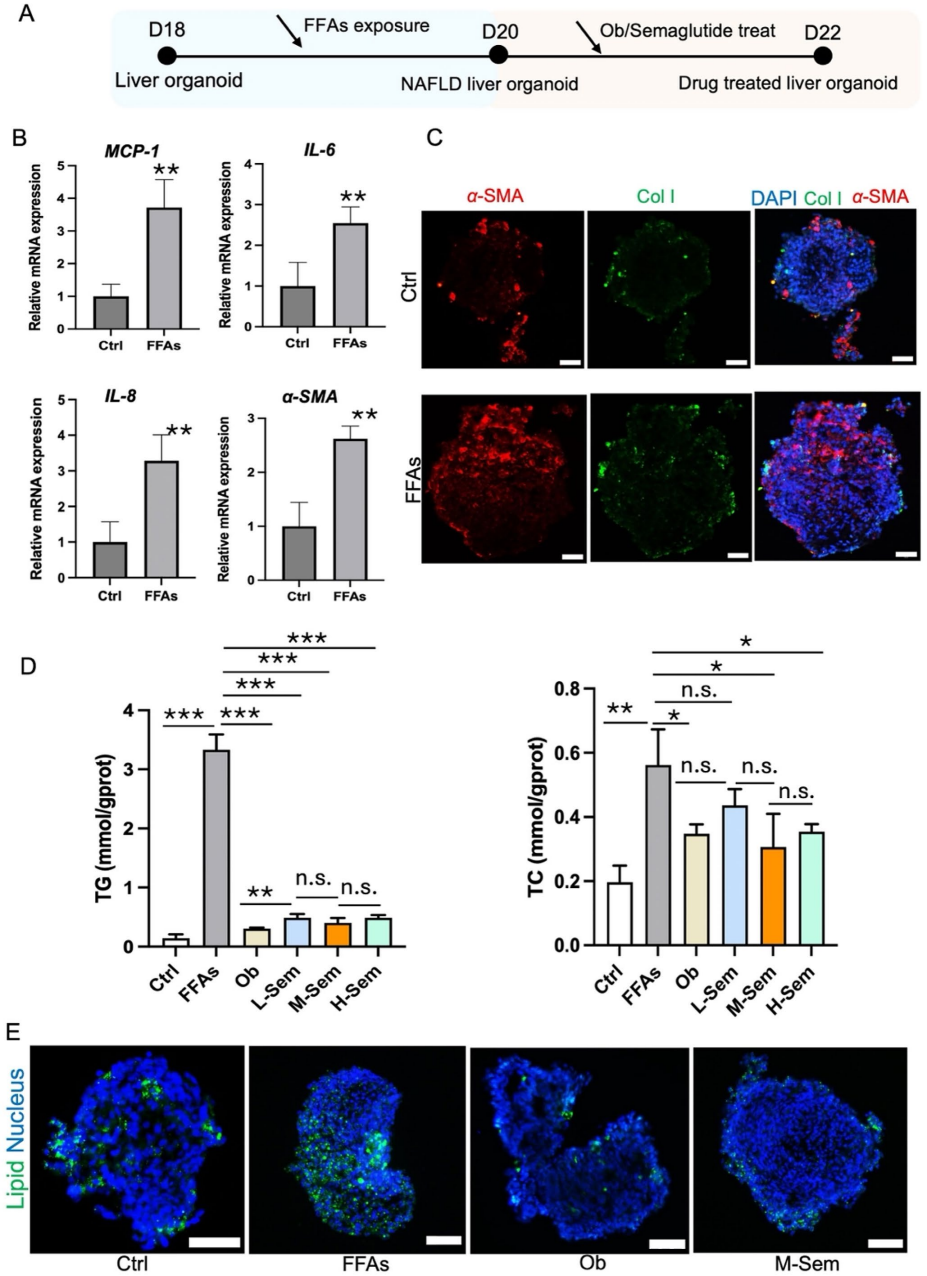
NAFLD modelling and semaglutide efficacy screening in hLOs. (A) Schematic representation of NAFLD induction protocol using free fatty acids (FFAs) and subsequent therapeutic intervention with obeticholic acid (Ob) or semaglutide. (B) RT‐qPCR quantification of inflammatory mediators (*MCP‐1*, *IL‐6*, *IL‐8*) and fibrosis marker (*α‐SMA*) expression during NAFLD progression. (C) Representative immunofluorescence micrographs showing α‐SMA and Col l distribution in control and FFA‐treated hLOs. (D) Quantitative assessment of triglyceride (TG) and total cholesterol (TC) levels following semaglutide treatment at varying concentrations. (E) BODIPY fluorescence analysis of lipid accumulation in cryosections from control, FFA‐treated, Ob‐treated and semaglutide‐treated (50 nM) hLOs. Scale bar: 50 μm. Data represent mean ± SD from three independent replicates (*p < 0.05, **p < 0.01, ***p < 0.001).

hLOs have emerged as a powerful tool in drug development and preclinical disease modelling, providing an effective platform for studying human liver pathophysiology [29]. A high‐fat diet is known to induce hepatic steatosis, which can trigger or exacerbate the progression of NAFLD [30], potentially leading to severe conditions such as liver fibrosis or cirrhosis. Currently, lifestyle modification remains the primary therapeutic strategy for NAFLD, underscoring the urgent need for novel therapeutic approaches or pharmacological interventions to mitigate the onset and progression of this condition. Semaglutide, a glucagon‐like peptide‐1 (GLP‐1) receptor agonist, has demonstrated promising therapeutic effects in animal models of high‐sugar and high‐fat diet‐induced liver disease, as well as in patients with obesity, diabetes and NAFLD [8]. Building on the previously described model, we further investigated the development of NAFLD under high‐fat media conditions. To simulate the transition from physiological to pathological states in hLOs, FFAs were introduced during the maturation phase (Day 18) for 2 days. Additionally, obeticholic acid (Ob), which has shown therapeutic efficacy in NAFLD‐associated hepatitis and fibrosis in several studies [31], was selected as the positive control drug, while semaglutide served as the test compound. Drug treatments were administered for 2 days following NAFLD induction to evaluate their therapeutic effects on NAFLD‐related injury (Figure 4A).

The accumulation of lipids in hepatocytes leads to lipid overload, which subsequently triggers the release of pro‐inflammatory cytokines [32]. First, we analysed lipid accumulation in physiological and pathological hLOs by immunofluorescence staining followed by percentage analysis, which was remarkable for the large accumulation of lipids (Figure S5). Then, to assess inflammatory and fibrotic responses in the system after FFA exposure, we analysed the expression of key markers. As shown in Figure 4B, compared to the untreated group, the relative mRNA levels of inflammatory cytokines (e.g., *MCP‐1*, *IL‐6* and *IL‐8*) and the fibrosis marker *α‐SMA* were significantly elevated in hLOs treated with FFAs, consistent with observations in human NAFLD. Furthermore, in the progression of NAFLD in vivo, increasing levels of inflammation cause hepatic fibrosis. Consistently, increased expression of the hepatic fibrosis marker α‐SMA was observed in FFA‐treated liver organoids, but no significant changes in collagen deposition were observed (Figure 4C). These findings suggest that 48 h of FFA treatment effectively mimicked the early stages of NAFLD, dominated by lipid accumulation and early inflammatory response, with elevated fibrosis markers and no formation of significant collagen deposition.

The hallmark of NAFLD is the abnormal accumulation of lipids, primarily triglycerides (TG) and total cholesterol (TC), within hepatocytes [33]. Therefore, TC and TG levels serve as direct indicators of altered hepatic lipid metabolism and are critical metrics for evaluating the lipid‐lowering efficacy of therapeutic agents. In this study, obeticholic acid (Ob) was used as the positive control, and NAFLD hLOs were treated with Ob (5 μM) and three concentrations of semaglutide (low: 20 nM, medium: 50 nM, high: 100 nM) for 2 days. As shown in Figure 4D, FFA exposure significantly increased TC and TG levels in hLOs, whereas treatment with the positive control Ob effectively reduced lipid accumulation. Notably, semaglutide exhibited favourable therapeutic effects across the tested concentration range (20–100 nM). The intermediate concentration (50 nM) of semaglutide demonstrated lipid‐lowering effects comparable to those of Ob, making it a suitable candidate for further evaluation. Triglyceride levels were 8‐fold lower and cholesterol levels were 1.8‐fold lower in M‐sem‐treated hLOs compared to the FFA‐treated group. Consequently, 50 nM semaglutide was selected for subsequent experiments to attenuate lipid accumulation. To further validate the therapeutic efficacy of 50 nM semaglutide, we performed immunofluorescence staining to assess lipid droplet accumulation. Compared to untreated hLOs, FFA exposure significantly increased lipid droplet aggregation, which was markedly reduced following treatment with both Ob and 50 nM semaglutide (Figure 4E). Collectively, these results demonstrate that the microporous array chip‐based hLO model provides a robust platform for in vitro drug screening and sensitivity testing in the context of liver pathophysiology.

### 3.5 Semaglutide Regulates Lipid Synthesis Catabolism, Inflammation and Fibrosis in NAFLD

Numerous studies have demonstrated that the onset and progression of NAFLD are accompanied by alterations in lipid synthesis and catabolism, as well as the secretion of inflammatory cytokines and the development of fibrosis [34]. To investigate the molecular mechanisms underlying semaglutide's therapeutic effects, we performed Kyoto Encyclopedia of Genes and Genomes (KEGG) and Gene Ontology (GO) enrichment analyses on differentially expressed genes (DEGs) between the FFA‐treated group and the semaglutide‐treated group (M‐Sem). The analysis was conducted using a threshold of *Q*‐value < 0.05 and |log_2_ fold change| > 1.

**FIGURE 5:**
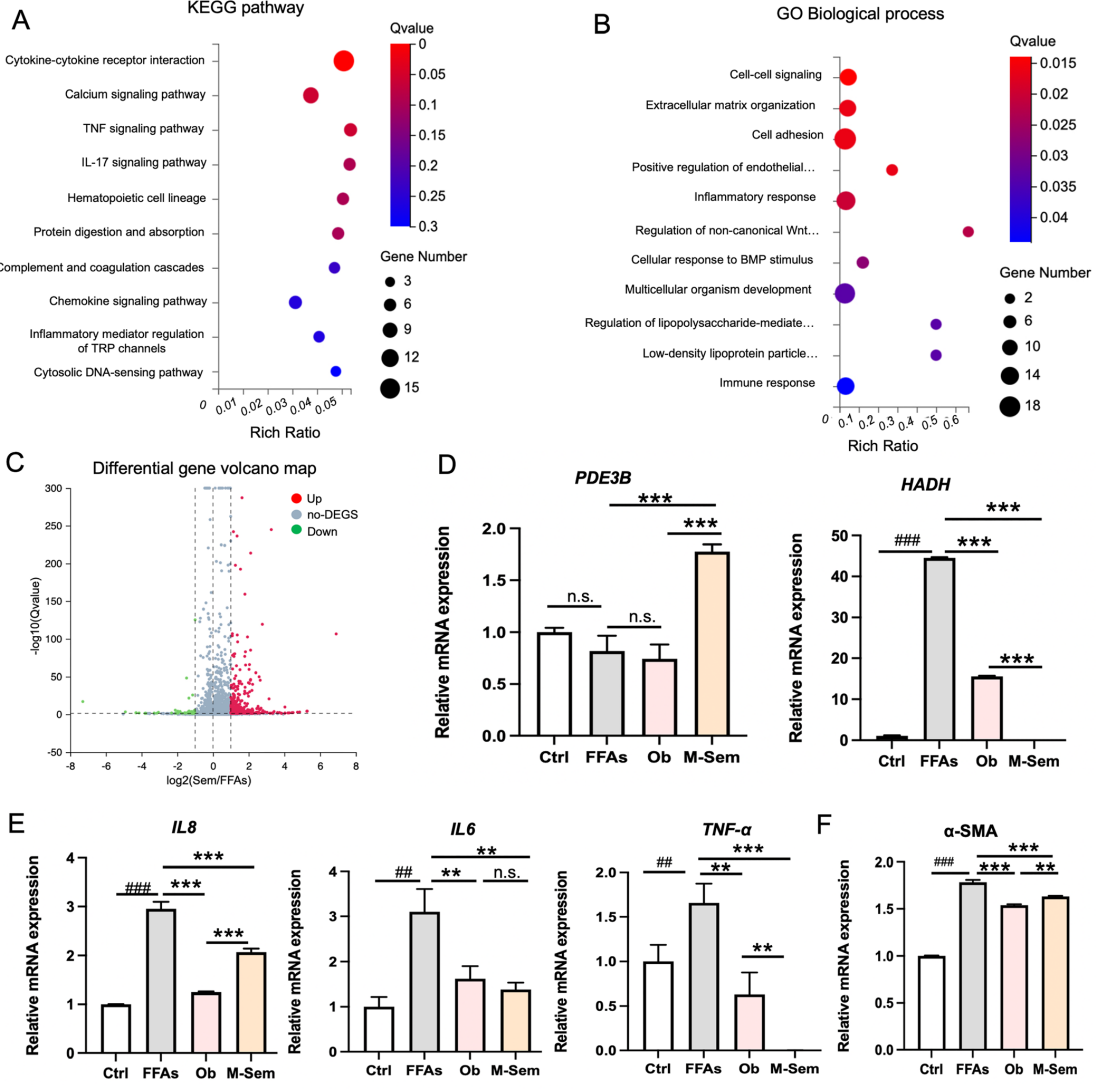
Transcriptomic and Molecular Analysis of Semaglutide's Effects in NAFLD Model. (A) KEGG pathway enrichment analysis highlighting modulation of inflammatory signalling networks in semaglutide‐treated versus FFA‐treated organoids. *N* = 3 replicates. (B) GO analysis revealing regulation of lipid metabolism pathways by semaglutide treatment. *N* = 3 replicates. (C) Volcano map analysis of differential genes. (D) Expression analysis of lipid metabolism regulators (PDE3B, HADH) under indicated conditions. (E) RT‐qPCR quantification of proinflammatory cytokines (IL‐8, IL‐6, TNF‐α) across treatment groups. (F) Quantification of fibrosis marker α‐SMA expression across experimental groups. Data represent mean ± SD from three independent replicates (*p < 0.05, **p < 0.01, ***p < 0.001 versus FFAs group).

KEGG pathway analysis revealed that the top 10 enriched pathways associated with DEGs were linked to inflammatory signalling pathways, including interleukin (IL), tumour necrosis factor (TNF) and inflammatory mediator regulation (Figure 5A). Similarly, GO analysis indicated that genes involved in lipid metabolism, such as those regulating low‐density lipoprotein (LDL) synthesis and catabolism, were significantly modulated following treatment with an intermediate concentration of semaglutide (Figure 5B). These findings align with previous reports suggesting that semaglutide regulates FFA‐induced biosynthetic processes. Meanwhile, we see 51 genes down‐regulated and 305 genes up‐regulated through the volcano plot (Figure 5C).

To further evaluate the therapeutic efficacy of semaglutide in NAFLD hLOs, we assessed key indicators related to lipid metabolism, inflammatory responses and fibrosis using real‐time PCR (Figure 5D–F). First, we examined the expression of phosphodiesterase 3B (*PDE3B*), a key enzyme involved in lipolysis. M‐Sem treatment significantly up‐regulated the PDE3B gene expression level by about 45% compared to the untreated group (Figure 5D). Additionally, hydroxyacyl‐coenzyme A dehydrogenase (*HADH*), which plays a critical role in fatty acid metabolism and energy production during lipid synthesis, was significantly downregulated after M‐Sem intervention, which was superior to that of obeticholic acid (Ob) (Figure 5D).

We also analysed the expression levels of pro‐inflammatory cytokines, including interleukin‐8 (*IL‐8*), interleukin‐6 (*IL‐6*) and tumour necrosis factor‐alpha (*TNF‐α*). Consistent with prior observations, FFAs induced elevated expression of these cytokines. Notably, semaglutide treatment markedly reduced their levels, demonstrating therapeutic effects comparable to those of Ob (Figure 5E). The pro‐inflammatory factors *IL‐8*, *IL‐6* and *TNF‐α* decreased approximately 1.5–2 fold after semaglutide intervention compared to the FFA group. Previous studies have shown that heightened inflammatory responses during NAFLD progression contribute to liver fibrosis [35]. In our study, we evaluated the expression of the fibrosis marker alpha‐smooth muscle actin (*α‐SMA*). FFAs significantly upregulated α‐SMA levels, while both Ob and semaglutide treatment alleviated this effect, indicating their potential anti‐fibrotic properties (Figure 5F).

In summary, the FFA‐induced liver organoid platform effectively recapitulates the transition from physiological to pathological states, characterised by dysregulated lipid metabolism, increased inflammatory factor secretion and fibrosis. These findings underscore the utility of the hLO platform for modelling the onset and progression of NAFLD in vitro. Importantly, semaglutide exhibits significant biological activities in mitigating the pathological hallmarks of NAFLD, including lipid‐lowering, anti‐inflammatory and anti‐fibrotic effects. These results provide valuable insights for the development of novel therapeutic strategies and functional drugs targeting NAFLD, as well as for informing clinical applications.

## 4 Conclusions

The microporous array chip‐based hLOs platform offers a substantive improvement for modelling NAFLD and for screening candidate therapeutics. By integrating stem cell biology, bioengineering and pharmacological analysis, we provide a robust framework for recapitulating NAFLD pathophysiology and evaluating drug efficacy in vitro. The liver organoids generated through this platform recapitulate key physiological characteristics of native hepatic tissue, including cholangiocyte differentiation, expression of mature liver‐specific markers, and the secretion of albumin and urea.

Furthermore, we successfully established a NAFLD model by treating the organoids with FFAs on the microarray chip, which elicited salient features of NAFLD such as lipid droplet formation, abnormal triglyceride accumulation and dysregulated lipid metabolism. Exposure to FFAs also led to the upregulation of inflammatory and fibrotic marker genes, thereby mirroring the pathophysiological events associated with NAFLD onset and progression. Subsequent intervention with semaglutide resulted in significant downregulation of triglyceride and total cholesterol accumulation, reduced lipid droplet formation, and amelioration of inflammatory and fibrotic responses, along with alterations in lipid synthesis and catabolism. These outcomes offer new insights into the underlying mechanisms of NAFLD and highlight the therapeutic potential of semaglutide in managing the disease.

Although we explored the therapeutic efficacy of Ob and semaglutide in NAFLD, the pathways of action of the two are distinct, and we did not explore the responses elicited by the joint action in NAFLD organoids. However, it is worth mentioning that Ob indirectly inhibits lipid synthesis mainly by pathways such as bile acid excretion, whereas semaglutide promotes lipolysis by reducing fat synthesis from scratch and FFA input [30, 36] Therefore, the mechanisms of the two should be complementary, and co‐administration may result in synergistic effects. A related study showed that the combination of GLP‐1 receptor agonists and FXR agonists reduced hepatic steatosis and fibrosis in a mouse model of non‐alcoholic steatohepatitis, which highlights the potential of co‐administration in metabolic liver disease [37].

Also, despite its innovations, our study has several limitations. First, the in vitro NAFLD model, while physiologically relevant, lacks systemic factors (e.g., immune cells, gut‐liver axis interactions) present in vivo [37]. Second, the short‐term FFA exposure (48 h) and drug treatment window may not fully capture chronic NAFLD progression or long‐term therapeutic outcomes. Third, the focus on semaglutide's lipid‐lowering effects overlooks potential off‐target or dose‐dependent toxicities, which require further investigation. Finally, while transcriptomics data highlighted key pathways and qPCR data validated, protein level analysis and metabolomics validation will strengthen the mechanism indirectly. Overall, the primary objective of our work is to present a user‐friendly human liver‐organoid‐on‐a‐chip platform with robust capabilities for studying organ development, disease modelling and drug screening. This system not only facilitates the investigation and validation of semaglutide's effects on NAFLD under pathological conditions but also supports a wide range of applications in preclinical drug development and mechanistic studies.

## Author Contributions

Conceptualization: Xiao‐yan You, Hui Wang and Guo‐ping Zhao. Investigation: Xiang‐yang Li. Methodology: Hui Wang. Supervision: Xiao‐yan You and Guo‐ping Zhao. Writing original draft: Xiang‐yang Li and Xiao‐yan You. Writing review and editing: Xiao‐yan You, Hui Wang and Guo‐ping Zhao.

## Ethics Statement

The authors have nothing to report.

## Conflicts of Interest

The authors declare no conflicts of interest.

## Supporting information


**Data S1:** cpr70118‐sup‐0001‐supinfo.docx.

## Data Availability

The data that support the findings of this study are openly available in the Supporting Information.
