# Supplementary material for: Development of NAFLD‐Specific Human Liver Organoid Models on a Microengineered Array Chip for Semaglutide Efficacy Evaluation
Source: Cell Prolif. 2025 Aug 27;59(4):e70118. doi: 10.1111/cpr.70118 (PMC13052169; doi:10.1111/cpr.70118)
Supplement: Supplementary file 1 — Data S1: cpr70118‐sup‐0001‐supinfo.docx. [file CPR-59-e70118-s001.docx]

**Supplementary Information**

**Development of NAFLD-specific human liver organoid models on a microengineered array chip for semaglutide efficacy evaluation**

**Authors**

Xiaoyan You ^a,b,c†*^, Xiangyang Li ^a,b,c,†^, Hui Wang ^b,d*^, Guoping Zhao ^b,e,f,g*^

**Institutional Affiliation**

^a^ Henan Engineering Research Center of Food Microbiology, College of food and bioengineering, Henan University of Science and Technology, Luoyang 471023, China

^b^ Master Lab for Innovative Application of Nature Products, National Center of Technology Innovation for Synthetic Biology, Tianjin Institute of Industrial Biotechnology, Chinese Academy of Sciences (CAS), Tianjin 300308, China

^c^ Haihe Laboratory of Synthetic Biology, Tianjin 300308, China

^d^ School of Life Sciences, Faculty of Medicine, Tianjin Key Laboratory of Function and Application of Biological Macromolecular Structures, Tianjin University; Tianjin, 300072, China.

^e^ CAS-Key Laboratory of Synthetic Biology, CAS Center for Excellence in Molecular Plant Sciences, Institute of Plant Physiology and Ecology, Chinese Academy of Sciences, Shanghai 200032, China

^f^ CAS Key Laboratory of Quantitative Engineering Biology, Shenzhen Institute of Synthetic Biology, Shenzhen Institute of Advanced Technology, Chinese Academy of Sciences, Shenzhen 518055, China

^g^ Engineering Laboratory for Nutrition, Shanghai Institute of Nutrition and Health, Chinese Academy of Sciences, Shanghai, 200031, PR China

†These authors contribute equally to this work

^*^Correspondence: [wanghuih@tju.edu.cn](mailto:wanghuih@tju.edu.cn) (H.W.), x[iaoyanyou@haust.edu.cn](mailto:xiaoyanyou@haust.edu.cn) (X.Y.), gpzhao@sibs.ac.cn (G.Z.)

**Supplementary Figures**


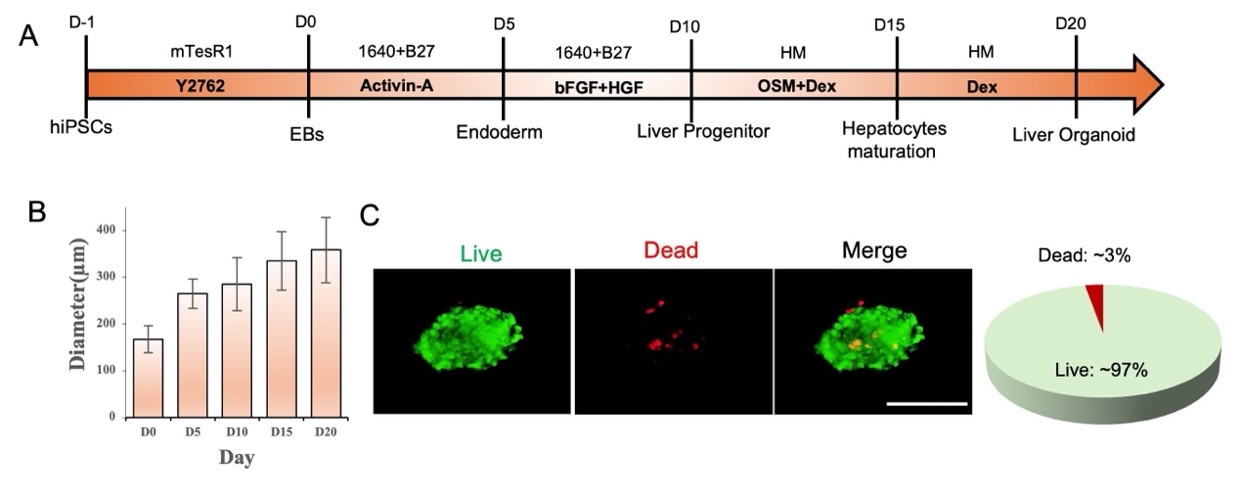


**Supplementary Figure 1.** (A) Flowchart of 3D liver organoid generation. (B) Mean diameter analysis of the sphere size distribution at each stage (n=15). (C) Cell viability of liver organoid at day 20 was assessed by live/dead kits and statistically analyzed. Scale bar: 200 µm.


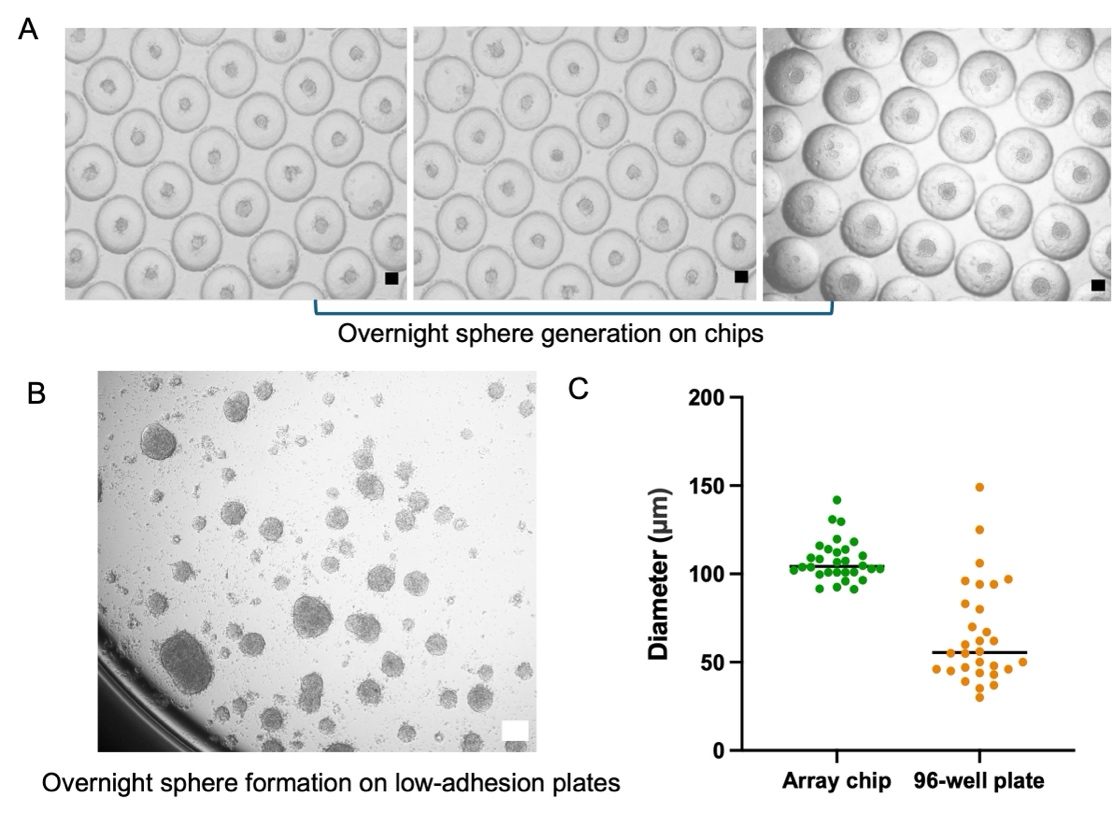


**Supplementary Figure 2.** (A) Spheroid formation after overnight incubation of hiPSCs inoculated into microporous array microarrays. Scale bar: 200 µm. (B) After overnight incubation, hiPSCs form spheroids in 96-well low-adhesion plates. Scale bar: 200 µm. (C) Compare and analyzed the size of the embryoid bodies formed in the two systems. (n=30).


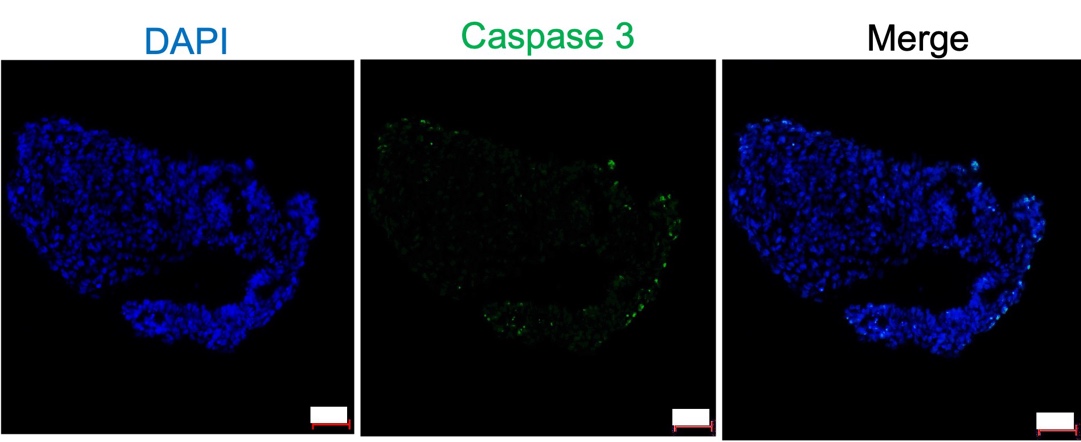


**Supplementary Figure 3.** Active caspase 3 immunostaining was performed on frozen sections at day 20 to show apoptotic cells (green) within liver organoid. Scale bar: 50µm.


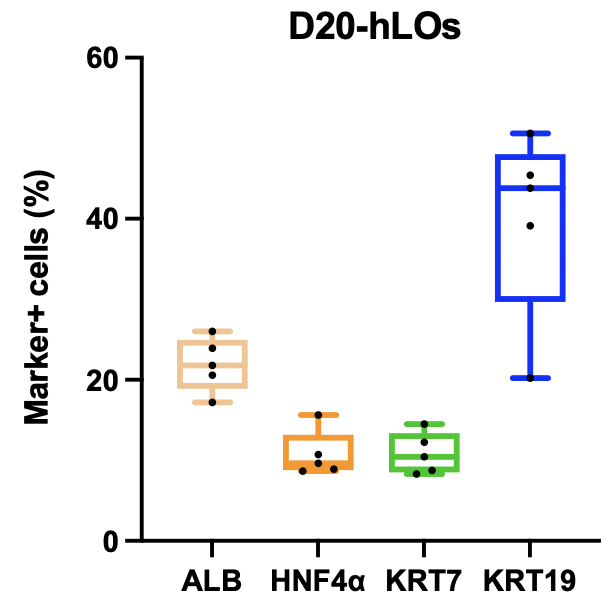


**Supplementary Figure 4.** The expressions of ALB, HNF4a, KRT7 and KRT19 were identified by immunohistochemical analysis and quantifications for the percentage of ALB^+^, HNF4a^+^, KRT7^+^ and KRT19^+^ cells in liver organoids. (n=5)


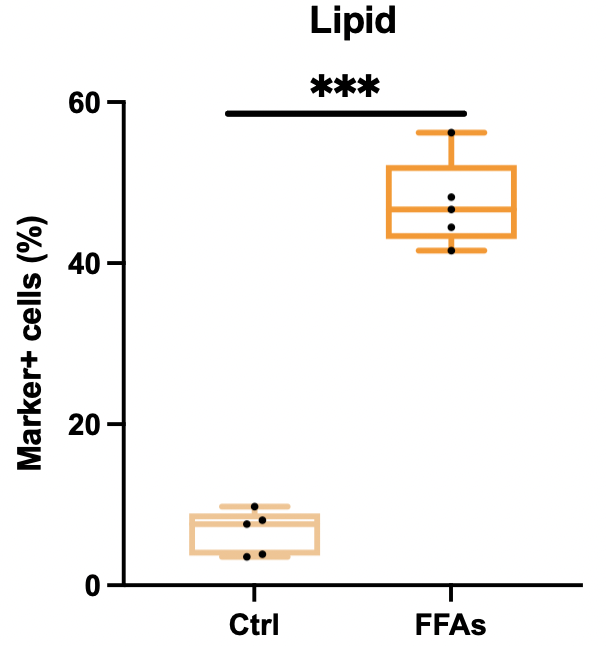


**Supplementary Figure 5.** Percentage analysis of lipid accumulation BODIPY staining in liver organoids. (n=5) (**P* < 0.05, ***P* < 0.01, ****P* < 0.001).

**Supplementary Tables**

**Supplementary Table 1.** A list of reagents used in the culture of physiological and pathological liver organoid.

| **Name** | **Supplier** | **Reference** | **Final concentration** |
| --- | --- | --- | --- |
| Human activin-A | PeproTech | 120-14-10 | 100 ng/mL |
| Human bFGF | PeproTech | 100-18B | 10 ng/mL |
| Human HGF protein | PeproTech | HZ-1084 | 20 ng/mL |
| KOSR | Life | 10828028 | 1% (v/v) |
| B27 supplement | Gibco | 17504044 | 1% (v/v) |
| Oncostatin M (OSM) | PeproTech | 300-10-10 | 10 ng/mL |
| Dexamethasone (Dex) | Sigma-Aldrich | D4902 | 10^−7^ M |
| Oleate acid (OA) | Sigma-Aldrich | O7501 | 400 μM |
| Palmitate acid (PA) | Sigma-Aldrich | P9767 | 200 μM |
| Bull serum albumin (BSA, protease free, fatty acid free) | Sigma-Aldrich | SRE0098 | 10 % (w/v) |
| RPMI 1640 medium | Gibco | C11875500BT | 1X |
| Hepatocyte medium, HM | ScienCell^TM^ | 5201 | 1X |

**Supplementary Table 2.** Primary antibody information for immunohistochemistry.

| **Primary antibody information** | | | | |
| --- | --- | --- | --- | --- |
| **Target** | **Supplier** | **Reference** | **Species** | **Dilution** |
| NANOG | Proteintech | 14295-1-AP | Rabbit | 1:200 |
| OCT4 | Abcam | ab109183 | Mouse | 1:500 |
| SOX2 | Affinity | AF5140 | Rabbit | 1:500 |
| FOXA2 | Abcam | Ab108396 | Rabbit | 1:600 |
| SOX17 | Proteintech | 24903-1-AP | Rabbit | 1:200 |
| AFP | Abcam | Ab169552 | Rabbit | 1:250 |
| Ki67 | Abcam | Ab92742 | Rabbit | 1:1000 |
| ALB | Bethyl | A80-129A | Goat | 1:1000 |
| HNF4a | Abcam | Ab201460 | Rabbit | 1:2000 |
| KRT7 | Abcam | Ab181598 | Rabbit | 1:8000 |
| KRT19 | Santa Cruz | Sc-6278 | Mouse | 1:500 |
| α-SMA | Abcam | Ab7817 | Mouse | 1:500 |
| Col I | Proteintech | 14695-1-AP | Rabbit | 1:2000 |
| AAT | Proteintech | 66135-1-lg | Mouse | 1:500 |
| CYP3A4 | Proteintech | 67110-1-lg | Mouse | 1:500 |
| Caspase 3 | Proteintech | 82202-1-RR | Rabbit | 1:500 |

**Supplementary Table 3.** Primer pairs for RT-PCR.

| **Primer** | **Forward sequence (5′→3′)** | **Reverse sequence (5′→3′)** |
| --- | --- | --- |
| GAPDH | CAATGACCCCTTCATTGACC | GACAAGCTTCCCGTTCTCAG |
| OCT4 | GGAGAAGCTGGAGCAAAACC | TGGCTGAATACCTTCCCAAA |
| NANOG | GATTTGTGGGCCTGAAGAAA | CTTTGGGACTGGTGGAAGAA |
| AFP | CTTTGGGCTGCTCGCTATGA | GCATGTTGATTTAACAAGCTGCT |
| TNF-α | ATGAGCACTGAAAGCATGATCC | GAGGGCTGATTAGAGAGAGGTC |
| IL8 | TTCTAGGACAAGAGCCAGGAAG | GGGTGGAAAGGTTTGGAGTATG |
| α-SMA | ATCATCACCAACTGGGACGAC | TTTCTCCCGGTTGGCCTTAG |
| PDE3B | AAAGGGGATAGAAAACTTAACAAGG | CAGGTAGCAATCCTGAAGTTCC |
| IL-6 | GGTACATCCTCGACGGCATCT | GTGCCTCTTTGCTGCTTTCAC |
| PLIN2 | GGGCAAAAGATGCTATGACG | TCAGTGAGAGGGAGGTACTGG |
| CPT2 | TGATGGTTGAGTGCTCCAAGT | AAGTGTCGGTCAAAGCCCTG |
| MCP-1 | CAGCAGCAAGTGTCCCAAAG | GAGTGAGTGTTCAAGTCTTCGG |
| CK7 | AAGAACCAGCGTGCCAAGTT | CACGCTCATGAGTTCCTGGT |
